# Supplementary material for: Causal Relationship between Aging and Anorexia Nervosa: A White-Matter-Microstructure-Mediated Mendelian Randomization Analysis
Source: Biomedicines. 2024 Aug 16;12(8):1874. doi: 10.3390/biomedicines12081874 (PMC11351342; doi:10.3390/biomedicines12081874)
Supplement: Supplementary file 1 [file biomedicines-12-01874-s001.zip › Supplementary Figure/Figure S8/Leaveoneout plot of MR analysis for TL to ubm-b-1655.pdf]

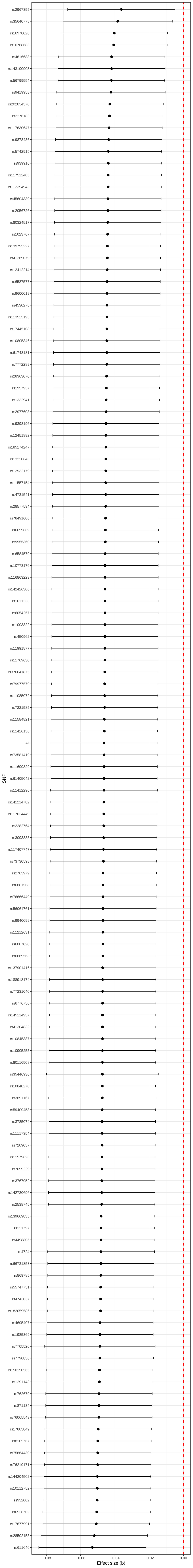
rs2967355
rs35640778
rs16978028
rs10768683
rs4616688
rs143190905
rs56799554
rs9419958
rs202034370
rs2276182
rs117630647
rs9878436
rs5742915
rs939916
rs117512405
rs112394943
rs45604339
rs2056726
rs80324517
rs1023767
rs139795227
rs41269079
rs12412214
rs6587577
rs9600019
rs4530278
rs113525195
rs17445108
rs10805346
rs61748181
rs7772289
rs28363070
rs1957937
rs1332941
rs2977608
rs9398196
rs12451892
rs185174247
rs13230646
rs12932179
rs11557154
rs4731541
rs28577594
rs78491606
rs6659669
rs9955360
rs6584579
rs10773176
rs116863223
rs142426306
rs1611236
rs6054257
rs1003322
rs450962
rs11991877
rs11769630
rs376641875
rs79977579
rs11085072
rs7221585
rs11584821
rs11426156
All
rs73581419
rs11699829
rs61405042
rs11412296
rs141214782
rs117034449
rs2282764
rs3093888
rs117407747
rs73730598
rs2763979
rs6881568
rs76666449
rs56061761
rs9940099
rs11212631
rs6007020
rs6669563
rs137901416
rs188918174
rs77231040
rs6776756
rs145114957
rs41304832
rs10845387
rs10905255
rs80116508
rs35446936
rs10840270
rs3891167
rs59409453
rs3785074
rs11117354
rs7209057
rs11579626
rs7099229
rs3767952
rs142730696
rs2538745
rs139669835
rs131797
rs4498805
rs4724
rs66731853
rs869785
rs55747751
rs4743037
rs182059586
rs4695407
rs1985369
rs7705526
rs7790856
rs150150565
rs1291143
rs762679
rs871134
rs76065543
rs17803849
rs8105767
rs75664430
rs76219171
rs144204502
rs10112752
rs932002
rs6536702
rs17677991
rs28502153
rs611646
SNP
-0.08
-0.06
-0.04
-0.02
0.00
Effect size (b)
